# Volumetric modulated arc therapy for lung stereotactic radiation therapy can achieve high local control rates

**DOI:** 10.1186/s13014-014-0243-1

**Published:** 2014-11-11

**Authors:** Hideomi Yamashita, Akihiro Haga, Wataru Takahashi, Ryousuke Takenaka, Toshikazu Imae, Shigeharu Takenaka, Keiichi Nakagawa

**Affiliations:** Department of Radiology, The University of Tokyo Hospital, 7-3-1 Hongo, Bunkyo-ku, Tokyo, 113-8655 Japan

**Keywords:** Primary lung cancer, Metastatic lung cancer, Stereotactic body radiation therapy, IMRT, VMAT

## Abstract

**Purpose:**

The aim of this study was to report the outcome of primary or metastatic lung cancer patients undergoing volumetric modulated arc therapy for stereotactic body radiation therapy (VMAT-SBRT).

**Methods and materials:**

From October 2010 to December 2013, consecutive 67 lung cancer patients received single-arc VMAT-SBRT using an Elekta-synergy system. All patients were treated with an abdominal compressor. The gross tumor volumes were contoured on 10 respiratory phases computed tomography (CT) datasets from 4-dimensional (4D) CT and merged into internal target volumes (ITVs). The planning target volume (PTV) margin was isotropically taken as 5 mm. Treatment was performed with a D95 prescription of 50 Gy (43 cases) or 55 Gy (12 cases) in 4 fractions for peripheral tumor or 56 Gy in 7 fractions (12 cases) for central tumor.

**Results:**

Among the 67 patients, the median age was 73 years (range, 59–95 years). Of the patients, male was 72% and female 28%. The median Karnofsky performance status was 90-100% in 39 cases (58%) and 80-90% in 20 cases (30%). The median follow-up was 267 days (range, 40–1162 days). Tissue diagnosis was performed in 41 patients (61%). There were T1 primary lung tumor in 42 patients (T1a in 28 patients, T1b in 14 patients), T2 in 6 patients, three T3 in 3 patients, and metastatic lung tumor in 16 patients. The median mean lung dose was 6.87 Gy (range, 2.5-15 Gy). Six patients (9%) developed radiation pneumonitis required by steroid administration. Actuarial local control rate were 100% and 100% at 1 year, 92% and 75% at 2 years, and 92% and 75% at 3 years in primary and metastatic lung cancer, respectively (*p* = 0.59). Overall survival rate was 83% and 84% at 1 year, 76% and 53% at 2 years, and 46% and 20% at 3 years in primary and metastatic lung cancer, respectively (*p* = 0.12).

**Conclusions:**

Use of VMAT-based delivery of SBRT in primary in metastatic lung tumors demonstrates high local control rates and low risk of normal tissue complications.

## Introduction

Stereotactic body radiation therapy (SBRT) is emerging as an effective treatment for patients with medically inoperable, early stage lung cancers as evidenced by local control rates reaching 90% at three years with relatively minimal toxicity [1,2].

The report from Mayo Clinic to validate the use of SBRT of 50 Gy in 5 fractions using IMRT 7 non-coplanar beams for 26 patients with medically inoperable Stage I lung cancer was published [3]. Use of IMRT during SBRT has not been without issues for some authors, with questions on the feasibility of IMRT delivery within small fields typical of SBRT [4], and concerns that organ motion could negate the benefits of the IMRT [5]. Recently, it was reported that volumetric modulated arc therapy (VMAT), which is a novel rotational technique and an extension of intensity modulated radiation therapy (IMRT), is applicable for SBRT for lung tumors [6-8]. This technique achieves treatment plan qualities comparable to the non-coplanar IMRT technique and dramatically decreases the total treatment time for each fraction [9].

To date, there are no published clinical studies specially addressing VMAT during SBRT for lung cancer. The purpose of the present report is therefore to provide clinical results for a group of primary or metastatic lung cancer patients whom we have treated with a VMAT-SBRT approach. This study has been done retrospectively and was proven by a local ethic/IRB board (No. 3372).

## Materials and methods

Tumor biopsy was attempted in all patients to confirm malignancy unless medically or surgically contraindicated. Before initiation of treatment, patients were expected to undergo complete history taking and physical examination, contrast-enhanced computed tomography (CT) imaging of the chest, [^18^ F] fluorodeoxyglucose positron emission tomography (FDG-PET) scanning for staging, pulmonary function testing (PFTs) including forced expiratory volume at 1 s (FEV1.0), and diffusion capacity to carbon monoxide (DLCO) only before SBRT and brain imaging (contrast-enhanced CT or magnetic resonance imaging [MRI]). The PFT was not performed routinely after SBRT.

### Patient and tumor characteristics

From October 2010 to December 2013, consecutive 67 lung cancer patients received single-arc VMAT-SBRT using an Elekta-synergy system. Complete data were acquired and evaluated for all 67 patients. Patient and tumor characteristics are summarized in Table 1. Among the 67 patients, the median age was 73 years (range, 59–95 years). Of the patients, male was 72% and female 28%. The median Karnofsky performance status was 90-100% in 39 cases (58%) and 80-90% in 20 cases (30%). Tissue diagnosis was performed in 41 patients (61%). There were T1 primary lung tumor in 42 patients (T1a in 28 patients, T1b in 14 patients), T2 in 6 patients, three T3 in 3 patients, and metastatic lung tumor in 16 patients. In our institution, some T3 tumors only with direct invasion to chest wall, without lymph node metastasis, and without the indication of radical surgery were treated with SBRT-alone. As to histology, squamous cell carcinoma was diagnosed in 26 cases, adenocarcinoma in 10 cases, small cell carcinoma in 2 cases, and non-small cell carcinoma, carcinoma, and undifferentiated carcinoma in one case, respectively.

**Table 1:** **Patient and tumor characteristics**

**Factors**	**No.**	**Rate**
Age		
Range	56-95 years old	
Median	73 years old	
Sex		
Male	48	72%
Female	19	28%
K-PS		
90-100%	39	58%
80-90%	20	30%
70%	2	3%
Unknown	6	9%
Smoking history		
With	29	43%
Without	17	25%
Unknown	21	31%
T stage		
T1a	28	42%
T1b	14	21%
T2	6	9%
T3	3	4%
Metastatic	16	24%
Pathological confirmation		
Without	26	39%
With	41	61%
Maximum diameter of primary tumor		
Range	7-51 mm	
Median	20 mm	
Tumor location		
Left upper	18	27%
Left lower	10	15%
Right upper	17	25%
Right middle	9	13%
Right lower	13	19%
Combined chemotherapy		
Both before and after SBRT	2	3%
Before SBRT	5	7%
After SBRT	3	4%
None	57	85%

39% of the patients were treated without histology. In primary cases without histology, PET/CT scan was compulsory. Either the SUV-max value over 3.0 by PET scan or the obvious tendency for increased tumor size by CT scans in the absence of significant up-take increase of FDG was necessary conditions for SBRT. Such cases had been discussed in weekly interdisciplinary cancer board and treatment decisions were based on recommendations of such a cancer board.

Selection of these patients for SBRT was not limited by baseline performance, pulmonary status, or tumor location. Patients were followed-up initially 6 to 8 weeks after completion of SBRT, with same-day PFTs and contrast-enhanced CT imaging of the chest. Thereafter, routine follow-up was every 3 months for 2 years, with CT imaging at each visit and PFTs twice yearly. Tumor responses were classified into complete response, partial response, stable disease, and progression based on Response Evaluation Criteria in Solid Tumors (RECIST). Toxicity was assessed retrospectively from patient clinical data using the Common Terminology Criteria for Adverse Events version 4.0 (CTCAE).

All patients underwent computed tomography (CT)-based SBRT planning for IMRT. Four-dimensional CT images for treatment planning were acquired with 2-mm-thick slices using an Aquilion LB model scanner (16-slice; Toshiba). The patients were in the supine position and fitted with an abdominal compressor [10,11]. The Elekta stereotactic body frame (SBF) was also used to minimize breathing artifacts for treatment planning CT. Scans were performed using the AZ-733 V system (Anzai Medical) as an external respiratory monitoring system. Each respiratory phase scan was transferred to a Pinnacle^3^, version 9.0, system (Philips).

The gross tumor volume (GTV) was delineated using the lung window (window, 1600 HU; level, −300 HU), on the 10 respiratory phase CT datasets from 4D CT respiratory sorting. Then, the ITV was produced using an integration of the GTVs as defined in these 10 phases. In all cases, the PTV was defined by adding a uniform 5-mm margin to the ITV to compensate for setup errors. Critical structures including but not limited to ipsilateral and contralateral lungs, spinal cord, and esophagus were outlined. PET-CT data were not used directly for image-guided planning.

Patients received a D95 prescription of 50 Gy (43 cases) before August 2013 or 55 Gy (12 cases) after August 2013 in 4 fractions for peripheral tumor or 56 Gy in 7 fractions (12 cases) for central tumor as defined by RTOG 0236 [12]. The single-arc VMAT-SBRT with 6 MV was created by SmartArc (Pinnacle^3^; Philips). Dose constraints for normal organs at risk for complications were the ipsilateral lung volume receiving 20 Gy (V20) <10% and 5 Gy <25% contralateral lung volume receiving 20 Gy (V20) <0% and 5 Gy <15%; spinal cord volumes receiving 15 Gy (V15) <0%; heart volumes receiving 30 Gy <0%; liver volume receiving 30 Gy <0%; body receiving 50 Gy <0%. Dosimetric planning and plan analysis were performed in Pinnacle^3^. The collapsed cone convolution method (which was comparable to superposition method) in Pinnacle^3^ was used as the heterogeneous correction method for the lungs. All final calculations were performed with a grid size of 2.0 mm. Dose distributions were calculated using peak exhalation CT data.

In primary cases, SBRT was the only treatment modality and no additional chemotherapy treatment was performed during disease control. In metastatic cases, no patients had received lung irradiation before SBRT.

Recently, a system for performing pre-treatment respiration-correlated CBCT, namely 4D CBCT, was developed by using an image-based recognition technique of the respiration phase [9,13]. Registration was semi-automatically performed using such pre-4D CBCT.

### Image guided RT procedure

VMAT-SBRT was performed using a photon beam provided by a Synergy linear accelerator (Elekta) equipped with the kV CBCT system that included a kV x-ray tube and flat-panel detector mounted on each side of the gantry, perpendicular to the treatment beam. In advance, the isocenter information of the treatment plan, regions of interest, and CT image set for peak exhalation were sent to the workstation with application software loaded onto the x-ray volume imaging functionality PC (XVI system, version 4.2). As an image guided RT (IGRT) procedure, pre-4D CBCT images were acquired with kV imaging parameters of a beam of 120 kVp and 20 mA/20 ms at an axial field length of 20 cm with a bow-tie filter immediately before daily treatment. In this case, the typical number of frames was approximately 650 in a pre-CBCT scan. Tumor registration was performed between obtaining a planning CT image for peak exhalation and obtaining the aforementioned 4D CBCT image. In the registration procedure, the chamfer matching (bone matching) was used first, and then, the manual registration was performed using ITV and PTV. Thereafter, the patient couch was adjusted according to the registration result.

### Statistical analysis

The outcomes of interest were local control and survival, measured from the time of the first day of SBRT until death or last patient contact. Local failure was defined as progressive and increasing CT scan abnormalities confirmed by progressive and incremental increases in the standardized uptake values (SUVs) of a lesion on serial PET imaging, with or without biopsy [14]. The survival probability was illustrated using Kaplan-Meier curves, and the log-rank test was used to determine whether a statistically significant difference was present among patient groups. The correlation coefficient was calculated between the occurrence of radiation pneumonitis required by steroid administration and the value of FEV1.0 or DLCO. The statistical analyses were performed using StatView 5.0 (SAS Institute, Cary, NC), and a value of p <0.05 was considered statistically significant.

## Results

### Results of SBRT planning

The median total treatment period was 6 days (range, 4–13 days). The ITV ranged from 1.6 to 80.1 cc (median, 5.8 cc). The median maximum, minimum, and mean dose within PTV was 58 Gy (range, 52.7-77 Gy), 48 Gy (range, 10.6-60.7 Gy), and 53.9 Gy (50.6-68 Gy). The median mean lung dose was 6.87 Gy (range, 2.5-15 Gy). The median lung V20, V15, and V5 were 9% (range, 3-21%), 12% (range, 5–30%), and 25% (9-60%), respectively.

### Clinical results

**Figure 1:**
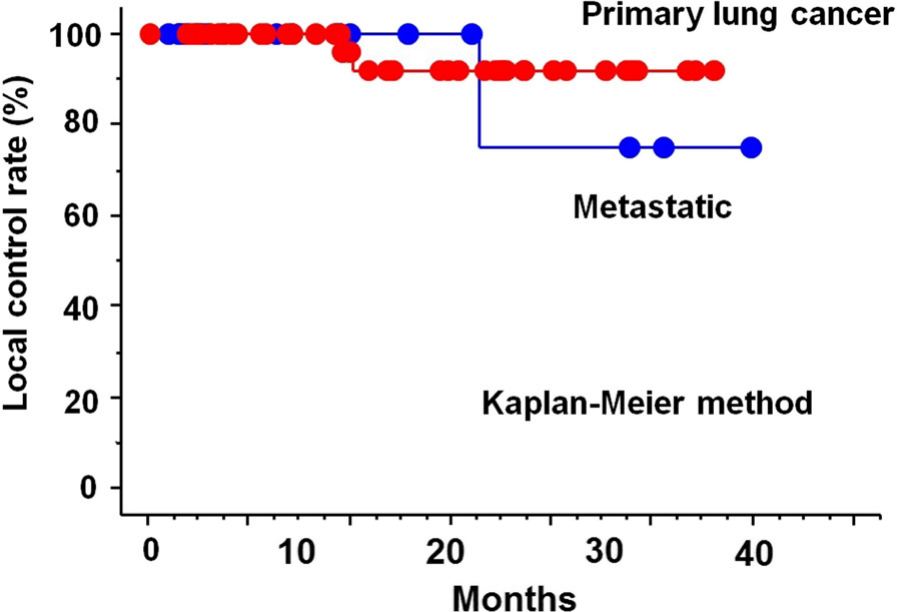
**Local control curves by primary and metastatic lung cancer.**

**Figure 2:**
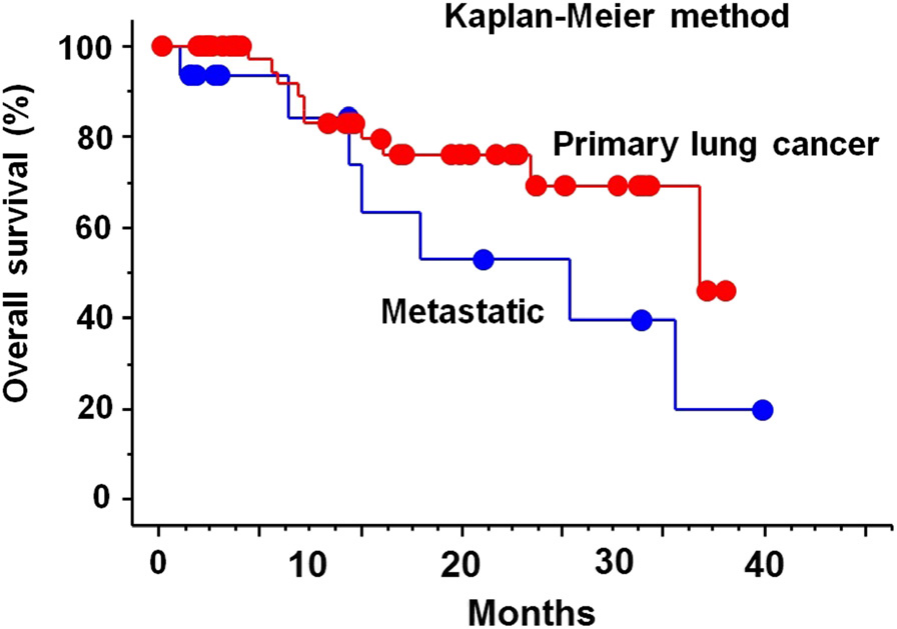
**Overall survival curves by primary and metastatic lung cancer.**

**Figure 3:**
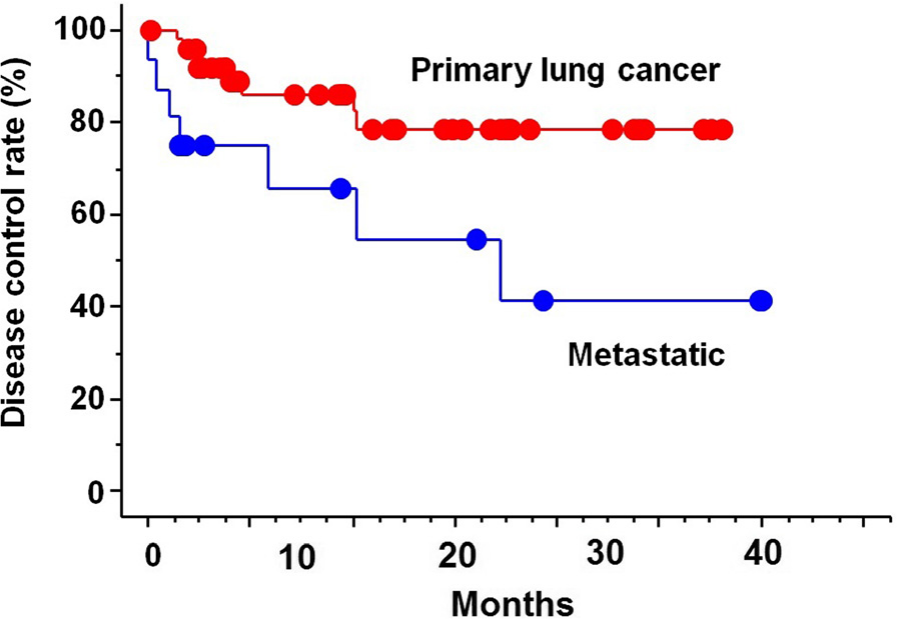
**Disease control curves by primary and metastatic lung cancer.**

The median follow-up was 8.9 months (range, 1.3-38.7 months). Limiting to surviving cases, the minimum follow-up time was 10.1 months. At the time of analysis, 48 cases were alive and 17 cases were dead. Actuarial local control rate were 100% and 100% at 1 year, 92% and 75% at 2 years, and 92% and 75% at 3 years in primary and metastatic lung cancer, respectively (*p* = 0.59) (Figure 1). Overall survival rate was 83% and 84% at 1 year, 76% and 53% at 2 years, and 46% and 20% at 3 years in primary and metastatic lung cancer, respectively (*p* = 0.12) (Figure 2). Actuarial disease control rate was 86% and 66% at 1 year, 78% and 41% at 2 years, and 78% and 41% at 3 years in primary and metastatic lung cancer, respectively (*p* = 0.013) (Figure 3).

For the dead patients of primary 10 cases and metastatic 7 cases, the cause of death was other metastases but lung in 11 cases, local problems in one case, and other disease in 5 cases. All three cases with primary T3 tumor were dead of distant metastases. For living 7 cases with disease (primary 4 cases and metastatic 3 cases), the pattern of relapse was all distant metastases.

### Toxicity

According to Common Terminology Criteria for Adverse Events (CTCAE) v4.0, grade 2 skin reactions were seen in 3 cases (grade 3 or more in zero) and grade 2 or more fatigue and chest wall pain requiring pain medication was never seen. Six patients (9%) developed radiation pneumonitis required by steroid administration. There was no correlation between the occurrence of radiation pneumonitis required by steroid administration and the value of FEV1.0 or DLCO. No other toxicities not lower than grade 3 were seen. No rib fractures or late esophageal toxicities were seen.

## Discussion

To our knowledge, this is the first report discussing the clinical result of IMRT SBRT using VMAT. One potential problem when inverse planning for lung cancer treatment is that the optimization of beam fluence only takes into account a single three-dimensional volumetric data set. For this, the actual VMAT that continuously delivers the dose in all respiratory phases may yield a dose distribution that differs from the plan. In order to compensate for this fact, the target volume can be extended to encompass the range of target motion by using 4D-CT scan, and field shapes can be defined as the target surroundings. Alternatively, the inverse plan that constrains MLC motion in VMAT forms field shapes that do not hide the target in lung cancer treatment. In this case, the beam intensity is mainly modulated by changing gantry speed and dose rate. Recently, it was reported that VMAT, which is a novel rotational technique and an extension of IMRT, is applicable for SBRT for lung tumors [7,15]. This technique achieves treatment plan qualities comparable to the non-coplanar IMRT technique and dramatically decreases the total treatment time for each fraction. VMAT drastically reduced the amount of radiation intensity, known as the monitor unit (MU). Thus, VMAT was found to be able to deliver the desired dose distribution in a shorter time than IMRT. Nowadays, VMAT plays a main role in high-precision radiotherapy treatment.

The study by Crabtree *et al.* [16] compared prospective clinical trials of SBRT, sub-lobular resection, and radiofrequency ablation of three clinical trials and they concluded that, among appropriately matched patients, no difference was seen in early morbidity between sub-lobar resection and SBRT. Both articles by Andratschke *et al.* [17] and by Guckenberger *et al.* [18] presented data of a large number of patients with inoperable stage I non-small cell lung cancer concerning safety of treatment and patterns of failure. Andratschke *et al.* [17] reported that all 10 local failures were observed in patients with T2 tumors (n = 61), isolated regional recurrence was observed in 7.6% (n = 6), and the crude rate of distant progression was 20.7% (n = 22) and that no grade 3–5 acute toxicity was observed. Guckenberger *et al.* [18] reported that 3-year freedom from local progression was 79.6% and that radiation-induced pneumonitis grade 2 or higher was observed in 38 of 512 patients (7.4%) and grade 5 was documented in two patients (0.4%).

The present clinical results support VMAT-based IMRT-SBRT delivery for lung tumors. There have been previous nonclinical studies validating the appropriateness of IMRT in the setting of radiosurgery or SBRT. Benedict *et al.* [19] carried out a planning study on four brain small lesion cases to assess the potential for improved tumor coverage and normal tissue sparing using intensity-modulated stereotactic radiosurgery. These investigators demonstrated significant dosimetric improvements for the small, irregularly shaped lesions compared with the results of other techniques, with reductions in critical organ irradiation. Cardinale *et al.* [20] reported similar results favoring IMRT in their report on three SBRT techniques, namely, arcs, non-coplanar fixed fields, and IMRT. In an analysis of organ motion effects on IMRT treatments with segments of few monitor units. Seco *et al.* [21] concluded that for most clinical cases, any non-negligible effects of IMRT dose delivery may be clinically irrelevant when multiple beams are used. On the other hand, Dvorak *et al.* [22] studied the impact of IMRT and leaf width on SBRT for liver and lung lesions. They compared plans generated for 10 patients treated with multi-leaf collimator (MLC)–based conformal therapy to seven alternative plans including one for micro-MLC IMRT treatment as in the BrainLAB system. They found that no significant differences could be observed in terms of target conformity between standard and IMRT dose distributions. Videtic *et al.* [3] studied the use of SBRT of 50 Gy in 5 fractions using IMRT seven non-coplanar beams using a Novalis/BrainLAB system for medically inoperable 28 Stage I lung cancers of 26 patients. They reported that the 3-year local control for T1 (22 lesions) and T2 lesions (6 lesions) was 93.1% and 100%, respectively. Hodge *et al.* [23] reported that delivery of IG-SBRT via helical tomotherapy using extreme hypofractionation in patients with early stage medically inoperable non-small cell lung cancer is feasible and well tolerated.

The limitations of this study include its retrospective nature. Although the small sample size also represents a limitation of this study, the median follow-up of close to 8.9 months, along with the meticulous documentation of patient status, lend support to the conclusions drawn from this cohort. Although this is a general limitation of lung cancer in following up with CT, it was the difficulty to discriminate between lung tissue changes after SBRT (i.e. post-RT scars) and true recurrences (i.e. progress) in this study, too.

## Conclusion

Use of VMAT-based delivery of SBRT in primary in metastatic lung tumors demonstrates high local control rates and low risk of normal tissue complications.
